# A Review of Traditional Chinese Medicine, Buyang Huanwu Decoction for the Treatment of Cerebral Small Vessel Disease

**DOI:** 10.3389/fnins.2022.942188

**Published:** 2022-06-29

**Authors:** Liying Sun, Xuhui Ye, Linlin Wang, Junping Yu, Yan Wu, Minpeng Wang, Lihua Dai

**Affiliations:** Intensive Care Unit, Shidong Hospital, Shanghai, China

**Keywords:** Buyang Huanwu Decoction, cerebral small vessel disease, collaterals disease, pharmacodynamics, active component

## Abstract

Cerebral small vessel disease (CSVD) is often referred to as “collaterals disease” in traditional Chinese medicine (TCM), and commonly includes ischemic and hemorrhagic CSVD. TCM has a long history of treating CSVD and has demonstrated unique efficacy. Buyang Huanwu Decoction (BHD) is a classical TCM formula that has been used for the prevention and treatment of stroke for hundreds of years. BHD exerts its therapeutic effects on CSVD through a variety of mechanisms. In this review, the clinical and animal studies on BHD and CSVD were systematically introduced. In addition, the pharmacological mechanisms, active components, and clinical applications of BHD in the treatment of CSVD were reviewed. We believe that an in-depth understanding of BHD, its pharmacological mechanism, disease-drug interaction, and other aspects will help in laying the foundation for its development as a new therapeutic strategy for the treatment of CSVD.

## Introduction

Cerebral small vascular disease (CSVD) is a common neurological disease that has serious impact on the patient’s health. Due to the insidious onset of CSVD, it is easy to be ignored by patients and even clinicians (Liu D. et al., 2019). In recent years, with the advancement of research, rapid progress has been made with respect to the risk factors, pathogenesis, clinical manifestations, and evaluation of CSVD (Shi and Wardlaw, 2016). CSVD refers to a series of clinical, imaging, and pathological syndromes caused by various etiologies affecting the small arteries and their distal branches, arterioles, capillaries, and venules in the brain (Li et al., 2018). Based on the imaging characteristics, CSVD includes recent small subcortical infarction, vascularity-induced lacunar infarction and white matter hyperintensity, perivascular space, cerebral microbleed, and cerebral atrophy (Wardlaw et al., 2013). The pathogenesis of CSVD is complex and is considered to be a dynamic disorder of the whole brain. Abnormal neurovascular unit (NVU) function plays an important role in its pathogenesis (Caruso et al., 2019). The NVU is composed of neurons, astrocytes, vascular endothelial cells, pericycles, and vascular smooth muscle cells that interact to regulate fluid and nutrient entry into the stroma, regulate cerebral blood flow, maintain and repair myelin sheath, and clear metabolites to achieve normal cell function (Iadecola, 2017). Changes in the structure or function of NVU for any reason can lead to CSVD (Bailey et al., 2014). The common mechanisms include chronic cerebral ischemia and hypoperfusion, endothelial dysfunction, blood-brain barrier (BBB) damage, inter-tissue fluid reflux disorder, inflammatory response, and genetic factors (Hase et al., 2018). The treatment strategies for CSVD in Western medicine are still limited to the treatment of acute ischemic stroke (AIS), including the control of anti-platelet aggregation, statins, and risk factors. Thus, Western medicine lacks multi-target and comprehensive treatment for CSVD.

Traditional Chinese medicine (TCM) has been developed for thousands of years in China. A TCM formula is developed based on experience through repeated clinical practice. A TCM formula usually consists of multiple drugs which act on the body organs through multiple targets to exert a therapeutic role. This multichannel approach is consistent with the multifactorial pathological mechanism of CSVD (Guo et al., 2020). Emerging studies have suggested that a variety of TCM formulas have therapeutic effects on CSVD. *In vivo* animal experiments and clinical case-control studies have confirmed the effectiveness of TCM formulas in the treatment of CSVD (Li et al., 2019), and it is believed that TCM formulas could be used as an effective supplement with Western medicine for CSVD therapy.

## Cerebral Small Vessel Disease and Collaterals Disease

According to TCM, the pathogenesis of CSVD lies in the collaterals. The collaterals are widely distributed in the human body and have the functions of nurturing and perfusion. They are narrow in shape and are located at the end of the branches of the blood vessels. This also determines the physiological characteristics of the slow movement of qi and blood and the pathological characteristics of qi deficiency and stasis, which can cause collateral dystrophy and stasis that affect the circulation of qi and blood, and in turn lead to abnormal collateral qi and blood infiltration, resulting in collateral disease (Huang D. et al., 2020). Collateral disease can cause qi and blood dysfunction and even structural damage (Eker et al., 2019). Moreover, the theory of collateral disease in TCM states that long-term illness enters the collaterals. Thus, collateral disease is a disease of essential empty and out solid, qi-deficiency is based, blood stasis is symptoms, qi deficiency and blood stasis are important pathogenesis of CSVD. The course of CSVD is dynamic (Nighoghossian and Mechtouff, 2020), with hemorrhage or ischemia constantly appearing. Hemorrhage and ischemia can exist at the same time, leading to the complexity of CSVD stagnation and stasis with old and new lesions, multiple deficiency and excess, and uneven distribution of multiple disease locations of the disease state (Winship, 2015).

According to the principle of “dredging collaterals,” combined with the basic pathogenesis of CSVD of qi deficiency and blood stasis, the main treatment of this disease is to invigorate qi, promote blood circulation, and remove collaterals. Buyang Huanwu Decoction (BHD) was first formulated by Wang Qingren, a doctor of TCM in the Qing Dynasty in “Yilin Cuogai.” The main functions of BHD are promotion of blood circulation and removal of blood stasis, dredging collateral, and supplementing qi, which are suitable for CSVD treatment (Zhang et al., 2018). Since the 1980s, BHD has been widely used in the clinical treatment of ischemic/hemorrhagic stroke (Cui et al., 2015; Dou et al., 2018). Its main components include *Astragalus, Ligusticum, Peach kernel, Radix Paeoniae Rubra, Geosaurus, Carthami Flos*, and *Angelica sinensis. Astragalus* tonifies qi; *Ligusticum* warms meridians and unblocks collaterals, activates blood, and relieves pain; *Peach kernel* activates blood circulation and removes stasis; *Radix Paeoniae Rubra* clears heat and cools blood; *Geosaurus* activates collaterals; *Carthami Flos* dredges the meridians, qi, and blood; and *Angelica sinensis* restores vital energy and invigorates the blood. The composition of a single daily dose of BHD is as follows: *Astragalus* 120 g, *Angelica sinensis* 6 g, *Radix Paeoniae Rubra* 4.5 g, *Geosaurus* 3 g, *Ligusticum* 3 g, *Peach kernel* 3 g, and *Carthami Flos* 3 g. The combination of these seven drugs in BHD exerts the maximum therapeutic effect on “Collaterals disease” in biomedicine.

## Traditional Chinese Medicine Prescriptions in Treatment of Cerebral Small Vessel Disease Based on Syndrome Differentiation

There is no record of “CSVD” in ancient Chinese medicine books. In view of the relationship between kidney and brain fully described in TCM theories and ancient TCM books, many modern TCM experts believe that the occurrence of CSVD is inseparable from kidney. Meng and Huo (2016b) believed that kidney deficiency was the basic pathogenesis of CSVD because of the physiological interaction between kidney and brain, the mutual function of ups and down, and the inseparable structure of marrow and brain collateral (Meng and Huo, 2016b). In addition, it has been reported that the cognitive dysfunction of CSVD belongs to the syndrome of deficiency of origin and symptom of reality, which is based on deficiency of kidney and marrow and marked by blood stasis. Therefore, the treatment of CSVD should be filled with essence, nourishing kidney, blood, and Yin, removing stasis and dredging collaterals (Cao et al., 2018). Supported by the biochemical theory of kidney essence and brain marrow in TCM, Zhang et al. (2018) observed the positive intervention effect of kidney nourishing therapy on the cognitive dysfunction caused by CSVD and improved the cognitive level of CSVD patients. Liang et al. (2019) tried to treat CSVD by filling kidney essence and nourishing kidney qi. A total of 252 CSVD subjects were randomly assigned to the intervention group and the control group. Changes in memory and executive screening (MES) scores were observed 24 weeks after treatment. The results showed that the strategy of tonifying kidney and removing stasis could improve MES score of subjects and delay the evolution of CSVD patients from mild cognitive impairment (MCI) to severe cognitive impairment and eventually to dementia (Liang et al., 2019). Meng and Huo (2016a) treated CSVD with kidney-tonifying combined with nootropic drugs, and the results suggested that this method could improve the Montreal cognitive assessment scale (MoCA), microbleed an atomical rating scale, and age-related white matter changes scores of CSVD patients (Meng and Huo, 2016a).

Qi is the governor of blood, qi deficiency leads to slow blood movement, over time, resulting in blood stasis in the collaterals. Phlegm turbid blood stasis is also an important pathogenic factor of CSVD. Therefore, supplementing qi and promoting blood circulation, removing stasis and dredging collaterals are important strategies of treating CSVD. Zhao et al. (2015) treated CSVD with traditional formula “Tongshen Funao Pill.” This formula has the effects of replenishing qi and promoting blood circulation, and it was found that the formula can significantly reduce serum inflammatory factors and improve their cognitive ability in CSVD patients (Zhao et al., 2015). Chen and Li (2019) used Huayu Tongluo Decoction to treat MCI caused by CSVD. The results showed that this formula can significantly improve the executive ability, attention and orientation of patients. The mechanism may be that this formula alleviated the stenosis and occlusion of cerebral small blood vessels, protects the integrity of nerve myelin sheath and cerebral small blood vessel wall, promoted angiogenesis and improved cerebral perfusion after ischemia (Chen and Li, 2019). Zhou H. et al. (2019) reached a similar conclusion, believing that the method of removing stasis and clearing collaterals could improve MoCA and activity of daily living score of CSVD patients, and played a role in improving cognitive function of CSVD patients.

BHD also exerted a therapeutic role in treating CSVD for qi deficiency and blood stasis syndrome (Cai et al., 2007). Xie (2018) selected BHD to treat CSVD through the method of invigorating qi, promoting blood circulation, removing blood stasis and dredging collaterals. The results suggested that BHD could inhibit the chronic inflammatory response and protect the intima of blood vessels, moreover, BHD improved blood rheology, increased the microcirculation, and cerebral perfusion of brain tissue and the syndrome of qi deficiency and blood stasis in patients with CSVD (Xie, 2018). Donepezil has been shown to improve cognitive impairment and delay disease progression in patients with CSVD (Battle et al., 2021). Xue et al. (2019) found in a controlled study that BHD combined with donepezil could improve the cognitive function of CSVD patients better than donepezil alone.

Therefore, BHD has a potential therapeutic effect on CSVD, which can improve cognitive impairment and daily living behavior ability of patients. However, BHD is currently mainly used for the treatment of cerebrovascular diseases (CVD), and clinical studies on the treatment of CSVD are limited. The mechanism of BHD treatment for patients with CSVD needs to be further elaborated. Next, we will further introduce the active ingredients and pharmacological effects of major Chinese herbs in BHD, and further elaborate the potential mechanism of BHD treatment of CSVD through network pharmacology prediction.

## Pharmacological Effects on the Active Ingredients of Buyang Huanwu Decoction

### Astragalus

*Astragalus* is the root of the leguminous plant *Astragalus mongolicus*. It invigorates qi, and thus can be used to treat qi deficiency. Previous studies have shown that *Astragalus* exerts antitumor and immunoregulatory effects (Yu J. et al., 2021), and treats chronic heart failure (Xu et al., 2021), diabetic nephropathy (Zhao et al., 2021) and diabetes mellitus with depression (Wang W. K. et al., 2021). In addition, *Astragalus* has been used for the treatment of CVD as it reduces brain tissues ischemia/reperfusion (I/R) injury (Guo L. Y. et al., 2021; Lo et al., 2021) and post cerebral ischemic inflammatory activation (Dou et al., 2021), inhibits brain microvascular endothelial cell injury (Tang X. et al., 2021), protects against thrombolysis-induced hemorrhagic transformation in cerebral ischemia (Pan et al., 2020), prevents Aβ oligomers-induced memory impairment and hippocampal cell apoptosis (Wang X. et al., 2020), and promotes hippocampal neurogenesis (Ni et al., 2020).

Modern pharmacological studies suggest that the active ingredients of *Astragalus* include astragalus polysaccharide, astragalus saponins, flavonoids calycosin, 3-hydroxy-9,10-dimethoxy pterostane, and astragaloside I, III, V. Astragalus polysaccharide is the main pharmacologically active ingredient in *Astragalus*. Studies have reported that it can suppress mitochondrial damage-mediated apoptosis (Gao et al., 2021b), modulate gut microbiota (Zhuang et al., 2021), enhance immune response (Zhou et al., 2021), alleviate cognitive impairment and β-amyloid accumulation *via* Nrf2 pathway (Qin et al., 2020), enhance remyelination (Ye et al., 2021), inhibit the formation of cerebral thrombosis (Sun et al., 2020), protect neurons, and stabilize mitochondrial in a Parkinson disease (PD) mouse model (Liu et al., 2018), and thus exhibit mitochondrial and anti-aging activity (Li et al., 2012).

### Ligusticum

*Ligusticum* is a TCM plant with a wide range of pharmacological functions, and is often used to promote blood circulation and qi, dispel wind, and relieve pain. It is suitable for improving blood stasis, and has been used to treat rheumatic arthralgia. *Ligusticum* is a qi medicine in blood, with functions of relieving depression and accessing and relieving pain. In recent years, studies have suggested the *Ligusticum* exerts therapeutic effect on ischemic stroke by neurogenesis and maintaining the BBB (Yu B. et al., 2021). In addition, it demonstrates neuroprotective effects by promoting adult neurogenesis and inhibiting inflammation in the hippocampus of cerebral ischemia rats (Wang M. et al., 2020). *Ligusticum* attenuates hyperhomocysteinemia-induced Alzheimer-like pathologies in rats (Zhang et al., 2021) and acts against focal cerebral ischemia (Gu et al., 2020). Moreover, *Ligusticum* alleviates acute lung injury (Jiang et al., 2021), prevents liver fibrosis (Wu et al., 2021), and has an anti-tumor effect (Zhong et al., 2021).

The pharmacological effect of *Ligusticum* is attributed to many active ingredients. These include trimethylpyridine, butylphthalide, senkyunolide, ferulic acid, chrysophanic acid, and a variety of volatile oils. Ligusticum lactone may be the main pharmacological active component of *Ligusticum* and is one of the most widely studied compounds of *Ligusticum*. It ameliorates cognition deficits by regulating docosahexaenoic acid metabolism in APP/PS1 mice (Huang J. et al., 2020), protects vascular endothelial cells from oxidative stress and rescues atherosclerosis by activating multiple NRF2 downstream genes (Zhu et al., 2019), alleviates myocardial ischemia injury through inhibiting autophagy (Wang G. et al., 2018), and reduces atherosclerotic lesions by inhibiting over expression of nuclear factor-κB (NF-κB)-dependent adhesion molecules (Xiao et al., 2014).

### Peach Kernel

*Peach kernel* are dried mature seeds of *Prunus persica* (L.) Batsch or *Prunus Davidiana* (Carr.). It can activate blood, remove blood stasis, moisten bowel and relieve constipation, and can be used for the treatment of amenorrhea, lung carbuncle, bowel lump, fall injury, dryness constipation, cough, and asthma. Recent studies have also found that *peach kernel* can be used to treat diabetic nephropathy (Han J. et al., 2021), colon cancer (Cassiem and De Kock, 2019), glucocorticoid-induced ischemic necrosis of femoral head (Qi and Chen, 2009), and cerebral I/R injury rats by regulating ADORA2A degradation (Li L. L. et al., 2020).

Peach kernel oil is the main active ingredient of *peach kernel*. Studies have shown that *peach kernel* oil could significantly decrease the low-density lipoprotein cholesterol levels in serum and reduce the area of the aortic atherosclerotic lesions in ApoE knockout mice. In addition, peach kernel oil could down regulate the tissue factors protein levels to inhibit the formation of atherosclerotic plaque (Hao et al., 2019).

### Radix Paeoniae Rubra

*Radix Paeoniae Rubra* is the dry root of herbaceous peony, and has the effect of clearing heat and cooling blood, promoting blood circulation, and removing blood stasis. Studies have shown that *Radix Paeoniae Rubra* exerts anti-tumor effect (Xu et al., 2013), improves chronic inflammation disease (Li X. H. et al., 2020), ameliorates lupus nephritis and lupus nephritis (Wang W. et al., 2020), protects against myocardial ischemic injury (Ke et al., 2017), exerts neuroprotective effects on ischemia stroke mice (Luo et al., 2020), has anti-thrombotic effect (Xie et al., 2017), ameliorates focal cerebral ischemic in rats (Gu et al., 2016), and promotes recovery of neurological function of stroke convalescent patients (Zhang et al., 2020).

Pharmacological analysis suggests that *Radix Paeoniae Rubra* contains Paeoniflorin, new Paeoniflorin, Paeoniae a, Paeoniae B, and palmitic acid. Amongst these compounds, paeoniflorin is the most widely studied and is considered to be the most important pharmacological active component of *Radix Paeoniae Rubra*. Paeoniflorin is a monoterpene glycoside with neuroprotective effect, and exerts antidepressant effects through enhancement of neuronal Fibroblast growth factor-2 (FGF-2) by microglial inactivation (Cheng J. et al., 2021; Wang X. L. et al., 2021c). It is also effective in preventing prenatal stress-induced learning and memory impairment (Wang X. et al., 2021). In a rat stroke model, paeoniflorin repressed neuroinflammation and facilitated neurogenesis (Tang H. et al., 2021), protected against ischemic brain injury, inhibited apoptosis (Liu et al., 2021), reduced cerebral oxidative stress, improved white matter integrity in hypoxic brain injury (Yang et al., 2021), and attenuated early brain injury through reducing oxidative stress and neuronal apoptosis (Wang T. et al., 2020). In a mouse model of Alzheimer’s disease (AD), paeoniflorin exerted neuroprotective effects *via* activation of adenosine A receptor (Kong et al., 2020). In addition, it could attenuate impairment of spatial learning and hippocampal long-term potentiation (Liu S. C. et al., 2019) and improve vascular dementia in rats *via* modulation of cannabinoid receptor 2 (Jinglong et al., 2013; Luo et al., 2018).

### Geosaurus

*Geosaurus* was first proposed in Shennong Classic of Materia Medica. It has been used in combination with a variety of TCMs. Li Shizhen proposed in Compendium of Materia Medica that *Geosaurus* has the function of activating collaterals, promoting blood circulation, removing blood stasis, and preventing and treating CVD. Clinically, *Geosaurus* is mainly used for treating myocardial damage (Han et al., 2014), asthma (Li et al., 2009), AD (Ren et al., 2006), and AIS induced by middle-cerebral artery occlusion (Liu et al., 2012). In addition, *Geosaurus* has the function of protecting cerebral microvascular endothelial cells against oxygen-glucose deprivation reperfusion (Sun et al., 2019) and promoting peripheral nerve regeneration (Chang et al., 2011).

The study of the chemical composition of the *Geosaurus* helps to understand its pharmacological effects. Modern chemical analysis has found that it contains a variety of chemical components, including lumbrokinase, palmitic acid, linoleic acid, alanine, lysine, and hypoxanthine. The therapeutic effect of lumbrokinase is mainly focused on preventing ischemic stroke and secondary ischemic stroke (Cao et al., 2013). In addition, it has been reported that it exerts anti-thrombosis effect by inhibiting the expression of intercellular adhesion molecule 1 and decreasing the immunoreactions of P-selectin and E-selectin in ischemic lesion (Zhang et al., 2003; Ji et al., 2008), inhibits intrinsic coagulation pathway, and activates fibrinolysis *via* an increase of t-PA activity (Jin et al., 2000). Lumbrokinase also has protective effects on hippocampus apoptosis and hippocampal function (Huang et al., 2013).

### Carthami Flos

*Carthami Flos* is an annual composite plant. It has the effect of dredging the meridians, qi and blood, dispersing blood stasis, and relieving pain. It is used for the treatment of amenorrhea, dysmenorrhea, chest pain, stagnation and abdominal pain, and prickly pain in chest and flank. Currently, *Carthami Flos* is believed to have a variety of pharmacological effects, including the antioxidant effect related to the potential anti-aging properties (Satoh et al., 2004), suppression of neutrophilic lung inflammation (Kim et al., 2014), inhibitory effect on cancer cells (Wu et al., 2013), regulation of gastrointestinal motility functions (Kim et al., 2017), treatment of traumatic intracranial hematoma (Sun et al., 2009), protection of hippocampal neurons induced by hypoxia injury (Yu et al., 2018), and protective effects on cerebral I/R injury (Wan et al., 2021).

A number of chemical components have been isolated from *Carthami Flos*, including carthamin, precarthamin, safflow yellow A and B, safflomin A, chlorogenic acid and caffeic acid may be the main pharmacological component of *Carthami Flos*. It has been reported in the literature that carthamin improves cerebral ischemia-recycling investment by attenuating information and ferroptosis (Guo H. et al., 2021), ameliorates diabetes mellitus and its cardiovascular composites (Orgah et al., 2020), protects the heart against ischemia/recycling investment (Lu et al., 2019), exerts neuroprotective activities (Hiramatsu et al., 2009).

### Angelica Sinensis

*Angelica sinensis* is a perennial herb [*Angelica sinensis* (*Oliv*) Diels] belonging to the umbelliferae family. It consists of the dried roots of the plant. Its pharmacological effects include enriching blood, promoting blood circulation, regulation of painful menstruation, and relieving skin numbness. *Angelica sinensis* has been used for treating AIS (Han Y. et al., 2021), preventing neural injury and promoting neuronal survival after ischemic stroke (Cheng C. Y. et al., 2021; Luo et al., 2021), protecting against I/R injury in the hippocampus (Cheng C. Y. et al., 2020), reducing Aβ-induced memory impairment (Duan et al., 2016), promoting synaptic plasticity during cognitive recovery (Deng et al., 2015), treating depression (Shen et al., 2016), and inhibiting malignant brain tumor growth effect (Tsai et al., 2006).

Amongst the various components, the neutral oil content in *Angelica sinensis* was the highest, including ligustilide, ligustryllactone, butyl phthalide, angelica ketone, neoangelica lactone, and coniferol ferulate, which may play an important role in the therapeutic effect of *Angelica sinensis*. Ligustilide is one of the main active ingredients of *Angelica sinensis*. Pharmacological studies on ligustilide have focused on its therapeutic effects on ischemic stroke and cognitive impairment. Ligustilide improves aging-induced memory deficit by regulating mitochondrial related inflammation (Zhu et al., 2020). It also contributes to the neuroprotective effect on AD by inhibiting the insulin-like growth factor 1 pathway (Kuang et al., 2014) and alleviates neurotoxicity induced by Aβ25-35 (Gao et al., 2021a). With respect to its therapeutic effects of ischemic stroke, ligustilide protects neurons through a variety of mechanisms, including anti-inflammatory and anti-oxidant signaling pathways, inhibiting ischemia-re-perfusion damage to the ischemic brain, and ameliorating the permeability of the BBB after stroke (Wu et al., 2019).

In summary, BHD may reduce neuronal apoptosis by inhibiting I/R injury, promote angiogenesis, reduce the release of immune-inflammatory factors and NK cell aggregation, improve synaptic plasticity and other mechanisms to protect neurons. In the next section, we describe our attempt to apply network pharmacology to further explore the pharmacological mechanism of BHD therapy for CSVD.

## The Active Compounds of Buyang Huanwu Decoction in the Treatment of Cerebral Small Vessel Disease

**FIGURE 1:**
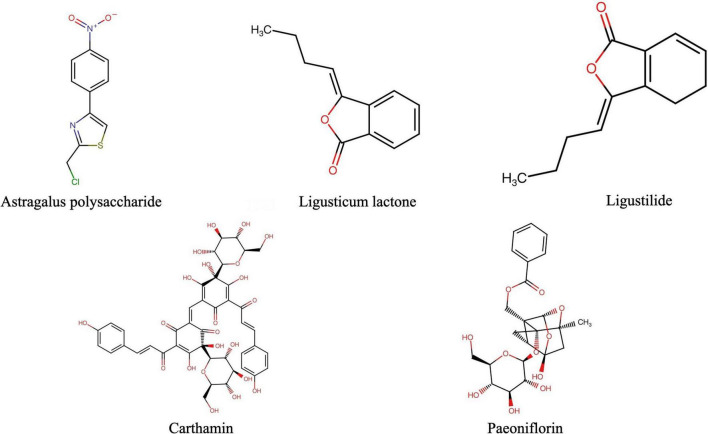
The 2D structures of major pharmacological active ingredients in BHD (BHD, Buyang Huanwu Decoction).

The anti-CSVD effect of BHD is a synergistic effect of multiple active ingredients, and involves the characteristics of multiple components, multiple targets, and multiple pathways. Network Pharmacology interprets the occurrence and development process of diseases from the perspective of system biology and biological network balance, understands the interaction between drugs and body from the holistic perspective of improving or restoring biological network balance, and guides the discovery of new drugs. With the increase in the research related to network pharmacology, it has been found that its characteristics of integrity and systematization are consistent with the holistic view of TCM and the principle of syndrome differentiation and treatment, and it has been widely used in the study of TCM. By searching the pharmacology database^1^ of the Traditional Chinese Medicine System Platform, the drug’s distribution, absorption, metabolism, excretion, and other parameters were initially screened, and we finally identified five most effective ingredients in BHD, including: astragalus polysaccharide, paeoniflorin, carthamin, and ligustilide. The Canonical SMILES of five effective components in BHD were obtained from PubChem;^2^ the 2-D structure was exhibited from the Swiss Target Prediction database (Figure 1). The Lab of Systems Pharmacology(see text footnote 1) and Swiss Target Prediction database^3^ were utilized to predict the target genes of the five effective components in BHD. The targets of CSVD and vascular dementia (VaD) were obtained from the MalaCards^4^, which is an integrated database of human maladies and their annotations, modeled on the architecture and richness of the popular GeneCards database of human genes(see text footnote 4) (Rappaport et al., 2017).

**FIGURE 2:**
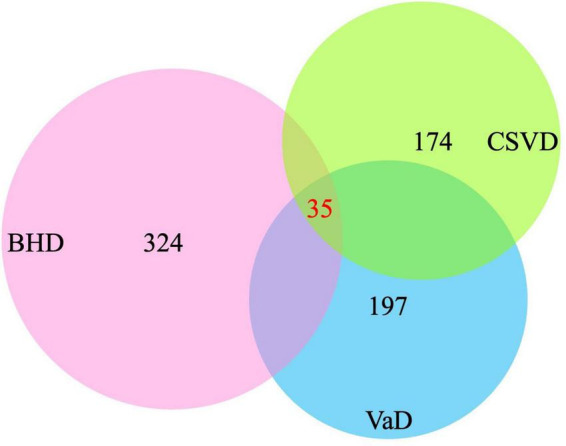
Venn diagrams was used to screen three groups of overlapping target genes, 35 collective targets of CSVD, VaD, and BHD were obtained (BHD, Buyang Huanwu Decoction; CSVD, Cerebral Small Vessel Disease; VaD, vascular dementia).

**FIGURE 3:**
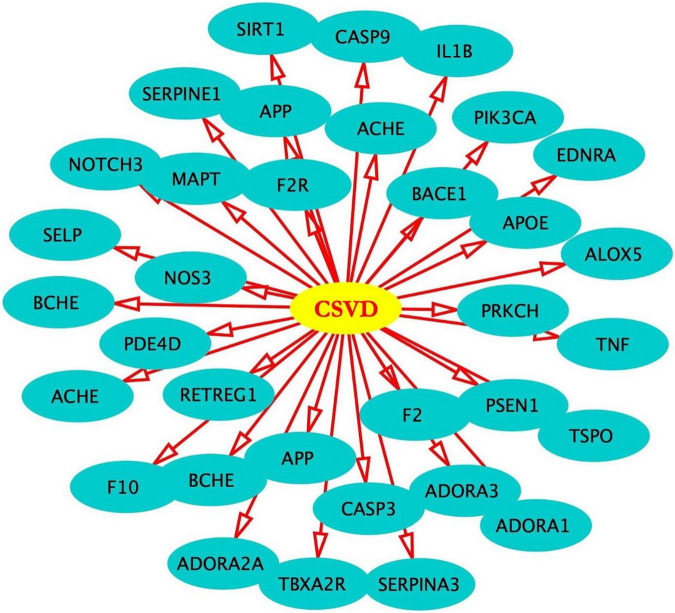
In total, 35 target genes were used to establish the regulatory network in Cytoscape.

**FIGURE 4:**
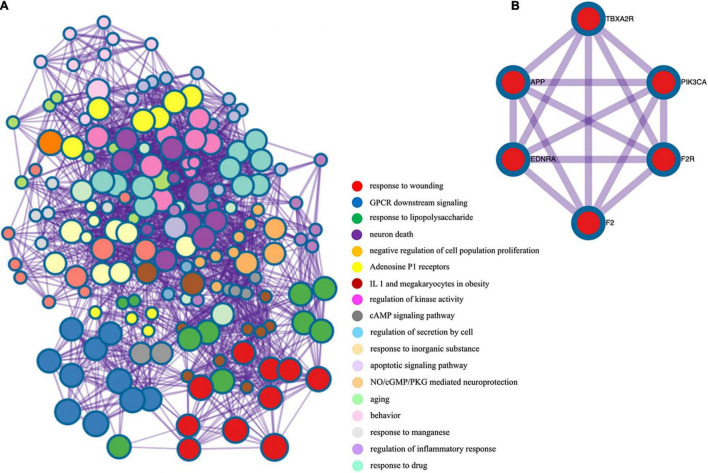
**(A)** Gene ontology enrichment analysis of overlapping target genes. Cross-examination of the relationship between these genes and GO biological process terms suggested that a substantial number of genes related to immune response were also enriched for other biological process such as defense response, response to cytokine stimulus and the inflammatory response. **(B)** Protein-protein interaction network and MCODE components identified in the gene list.

After screening, 324 target genes of BHD, 174 target genes of CSVD and 197 target genes of VaD were identified. Venn diagrams was used to screen two groups of overlapping target genes, 35 collective targets of CSVD, VaD and BHD were obtained (Figure 2). Using Metascape^5^, we further performed protein-protein interaction (PPI) analysis and interactive visualization of Gene Ontology (GO) networks for collective target genes (Zhou Y. et al., 2019). The PPI was constructed by Cytoscape (Figure 3), and the network of enriched terms was analyzed by Metascape (Figure 4). The top 10 clusters with representative enriched terms of BHD effects on CSVD are shown in Table 1. Therefore, BHD may have therapeutic effects on CSVD through these enriched pathways.

**TABLE 1:** Top 10 clusters with their representative enriched terms (one per cluster).

GO	Category	Description	Count	%	Log10(*P*)
GO:0009611	GO biological processes	Response to wounding	14	56	−17.42
GO:0032496	GO biological processes	Response to lipopolysaccharide	9	36	−11.05
GO:0070997	GO biological processes	Neuron death	9	36	−10.84
GO:0043549	GO biological processes	Regulation of kinase activity	9	36	−7.98
GO:1903530	GO biological processes	Regulation of secretion by cell	8	32	−7.7
GO:0010035	GO biological processes	Response to inorganic substance	8	32	−7.65
GO:0097190	GO biological processes	Apoptotic signaling pathway	8	32	−7.6
GO:0030162	GO biological processes	Regulation of proteolysis	8	32	−6.74
GO:0007610	GO biological processes	Behavior	7	28	−6.27
GO:0007568	GO biological processes	Aging	6	24	−6.37

*“Count” is the number of genes in the user-provided lists with membership in the given ontology term. “%” is the percentage of all of the user-provided genes that are found in the given ontology term. “Log10(P)” is the p-value in log base 10.*

## Conclusion and Prospect

CSVD is a clinical imaging syndrome that involves small cerebral vessels and is one of the main causes of vascular dementia. It includes hemorrhagic/ischemic and acute/chronic changes. The current treatment strategies lack multi-target and multi-mechanism approaches, which is one of the important factors for the unsatisfactory treatment effect of CSVD in recent years. However, with increased research on the pharmacological mechanisms of TCM, the effective ingredients of TCM have attracted increased attention. BHD is a famous TCM formula, which has the function of promoting blood circulation and removing blood stasis, dredging the meridians and collaterals. It can be used to treat various diseases caused by qi stagnation and blood stasis. At present, it is mainly used to treat various CVD, including ischemic/hemorrhagic diseases. Here, we systemically reviewed the effects of BHD on CVD in terms of its various components, pharmacological mechanisms, clinical studies, and effective components. We further performed PPI and GO pathway analyses to elaborate the possible pharmacological effects of BHD on CSVD.

Currently, BHD is widely used in treating CVD and exerts therapeutic effects with fewer side-effects. However, the active ingredients in BHD still need to be further explored, and the pharmacological mechanism of its treatment for CSVD needs to be clarified. In addition, although clinical studies on the combined application of BHD and aspirin have confirmed that BHD can improve the therapeutic effect and reduce the incidence of complications, it still needs to be further confirmed by well-designed large-scale clinical controlled studies. Network pharmacology provides a new platform to study the effective components and pharmacological effects of TCM. We believe that it will play an important role in promoting the discovery of new effective components in TCM formulas and in the modern application of TCM.

## Author Contributions

LS, XY, LW, JY, YW, and MW contributed to the literature search, data extraction, and data analysis. LD contributed to the project design and manuscript writing. All authors have read and approved the final version of the manuscript.

## Conflict of Interest

The authors declare that the research was conducted in the absence of any commercial or financial relationships that could be construed as a potential conflict of interest.

## Publisher’s Note

All claims expressed in this article are solely those of the authors and do not necessarily represent those of their affiliated organizations, or those of the publisher, the editors and the reviewers. Any product that may be evaluated in this article, or claim that may be made by its manufacturer, is not guaranteed or endorsed by the publisher.

## Footnotes

1
http://www.tcmspw.com/

2
https://pubchem.ncbi.nlm.nih.gov/

3
http://www.swisstargetprediction.ch/

4
https://www.malacards.org/

5
http://metascape.org
